# A Low-Cost Calibration Method for Low-Cost MEMS Accelerometers Based on 3D Printing

**DOI:** 10.3390/s20226454

**Published:** 2020-11-12

**Authors:** Jesús A. García, Evangelina Lara, Leocundo Aguilar

**Affiliations:** Facultad de Ciencias Químicas e Ingeniería, Universidad Autónoma de Baja California, Tijuana BC 22390, Mexico; garcia.jesus@uabc.edu.mx (J.A.G.); laguilar@uabc.edu.mx (L.A.)

**Keywords:** accelerometer, calibration, inertial sensor, mems, 3D print, low-cost

## Abstract

A ubiquitous sensor in embedded systems is the accelerometer, as it enables a range of applications. However, accelerometers experience nonlinearities in their outputs caused by error terms and axes misalignment. These errors are a major concern because, in applications such as navigations systems, they accumulate over time, degrading the position accuracy. Through a calibration procedure, the errors can be modeled and compensated. Many methods have been proposed; however, they require sophisticated equipment available only in laboratories, which makes them complex and expensive. In this article, a simple, practical, and low-cost calibration method is proposed. It uses a 3D printed polyhedron, benefiting from the popularisation and low-cost of 3D printing in the present day. Additionally, each polyhedron could hold as much as 14 sensors, which can be calibrated simultaneously. The method was performed with a low-cost sensor and it significantly reduced the root-mean-square error (RMSE) of the sensor output. The RMSE was compared with the reported in similar proposals, and our method resulted in higher performance. The proposal enables accelerometer calibration at low-cost, and anywhere and anytime, not only by experts in laboratories. Compensating the sensor’s inherent errors thus increases the accuracy of its output.

## 1. Introduction

In the Embedded world, a sensor that has reached a ubiquitous status is the accelerometer. Its small size, low-cost, and low power-consumption allow its integration in many consumer products, such as smart phones, health monitors, fitness trackers, gaming systems, etc. An accelerometer allows motion tracking, which has a wide range of applications, including indoor navigations systems, user interfaces responsive to human gestures, manoeuvre of unmanned aerial vehicles (UAVs), monitoring of training-load in sports, fall detection for seniors health care, etc. [1,2,3,4,5,6,7,8].

An inertial measurement unit (IMU) uses accelerometers to detect the linear acceleration of a body. An accelerometer consists of three orthogonally-mounted sensors that detect the acceleration in three axes: x, y, and z. IMUs are Micro-electro-mechanical systems (MEMS), a technology of micro electronic and precision machinery. However, due to the characteristics of the materials used in MEMS and their fabrication process, IMUs present errors that affect the accuracy of the acceleration measurements [9]. The types of error are deterministic and random, and can be grouped as bias, scale factor, and axes misalignment. Bias error is the measured acceleration when the sensor is subject to no acceleration, i.e., is the deviation from a zero measurement [4,10]. Scale factor is the sensibility of the sensor, which corresponds to the ratio of input–output changes. It is evaluated as the gradient of the best straight line fitted by the least squares method to the input versus output data. Axes misalignment is a mounting error in the fabrication process that results in non-orthogonal axes in the sensor body frame, which causes couplings in the sensor output signals [11]. These errors are a major concern in navigation systems that use inertial sensors. As they accumulate over time, they degrade the position accuracy, causing fast position drifts [4,12]. The errors can be modeled and compensated through a calibration process, in which the sensor output is compared with reference information to determine the coefficients that make them coincide, over a range of output data [10]. Many calibration methods for accelerometers have been proposed; however, they use sophisticated equipment to position the sensor in precise orientations to evaluate its output with the reference information, such as [13,14,15,16,17]. As the specialized equipment is usually available only in laboratories, the proposals are expensive and complex, which limits their widespread adoption [9].

In this article, a low-cost and practical calibration method is proposed. The target applications of the proposal are indoor navigation and pedestrian step-length estimation, and the physical environment where the accelerometer will be used is indoors. The calibration method can be used in applications that utilize inertial sensors for the guidance of people in airports and shopping malls, and for the guidance of firefighters inside buildings with low visibility due to smoke. The calibration method can also be applied in systems that use accelerometers for health status prediction and gait analysis [18]. The advantage of using inertial sensors to develop these applications is that they do not require the addition of infrastructure to the environment—contrary to other systems such as network-based systems, which estimate the user position using wireless signals such as Long-Term Evolution (LTE), Ultra Wide Band (UWB), Wi-Fi, and Bluetooth of access points deployed in the building [19]. The proposed calibration method consists of three simple steps that do not require sophisticated equipment for its execution. They perform a sanity check, a multi-position evaluation, and a stability test. The method is a gravity-based calibration, which means that it uses the gravity vector as reference information to determine the coefficients that compensate the accelerometer output. To be able to use gravity as reference, the sensor has to be placed in a precise set of orientations. A 3D printed polyhedron is used to accomplish the accurate positioning. Considering the popularization and availability of 3D printers in the present day, the polyhedron can be obtained at a low-cost. Additionally, each polyhedron could hold as much as 14 sensors at the same time, and all of them can be calibrated simultaneously. The absence of sophisticated tools allows for performing the calibration anywhere and anytime, not only by experts in laboratories. Furthermore, a mathematical model based in the Allan Variance [20,21] was used to estimate the velocity random walk and bias instability errors in the sensor output [22]. It is a simple model that requires low computation and provides precise noise terms present in inertial sensor data. The proposed method was performed with a low-cost sensor. The performance was evaluated by computing the RMSE of the accelerometer output before and after the calibration, resulting in a error reduction of 88.46%. The error was also compared with the reported in similar proposals, and our method resulted in higher performance.

The rest of the article is organized as follows: Section 2 contains Related work. Section 3 presents background information about the accelerometer error model. The proposed method is described in Section 4. The calibration with a low-cost accelerometer is presented in Section 5. Section 6 contains Discussion. Finally, Section 7 presents Conclusions.

## 2. Related Work

In this section, current proposals of accelerometer calibration are briefly analyzed.

A comparison of three gravity-based calibration methods is presented in [23]. The differences between them are the mathematical models used to obtain the error terms and the acceleration range applied to the sensor. Concerning the latter, one approach had a range of 0 g to 1 g on each axis, and the other two −1 g to 1 g. The methods were compared by computing their root-mean-square deviation (RMSD). The lowest obtained by the approaches were 0.031, 0.0215, and 0.0216. In applications that require high accuracy in the acceleration measurements, such as Inertial Navigation Systems, where the acceleration of a body is used for its position determination [24], those error values can degrade the performance of the system, as they accumulate over time causing fast position drifts [25].

Several methods that require positioning the sensor in precise orientations to estimate the calibration parameters have been proposed, such as [15,16], where robotic arms are used to control the sensor’s orientation and thus the influence of the gravity force on its axes. The proposal in [15] uses fixed positions, whereas in [16] the orientations are determined online during the calibration, aiming to decrease the variance in the axes with the largest uncertainty. In both proposals, acceleration measurements are recorded and different mathematical models are applied to estimate the noise terms, such as unscented Kalman filter [26] and least-squares optimization. Similarly, in proposals [14,17], a motion rate table and a three-axis turn-table are used to position the sensor, respectively; and Kalman filters are applied for the noise terms estimation. All these methods require the use of specialized equipment found only in laboratories. In addition, the number of accelerometers that can be calibrated at the same time is limited to the number of sensors that the robotic arms and controllable-motion tables can accommodate, which is usually a very low number or even only one. Because of this, the calibration is not low-cost and is complex in some cases. Moreover, it can only be performed where the equipment is located.

In [27], a two-degree-of-freedom (DOF) mechatronic structure is proposed to generate controlled linear and angular accelerations in the sensor. The structure includes shaft encoders and a microcontroller unit to obtain its position when in motion. The actual acceleration experienced by the sensor is computed using conventional kinematic equations, such as Euler angles, and it is contrasted with the sensor output. Additionally, filtering techniques are used in the measurements to eliminate noise terms caused by machining errors in the structure. However, the structure is large, requiring many space for its installation, and it is not portable, as it has to be carefully assembled and mounted to a wall. Similarly, the work in [28] proposes a machined metal frame to place the accelerometer in twelve positions to calibrate against gravity. However, our proposed figure is significantly cheaper to make because it is made of plastic. In addition, our figure has more angles of inclination to stimulate the sensor’s axes, which could provide a more precise calibration.

In this article, a simple calibration method is proposed. It does not require sophisticated equipment, as it only uses a 3D printed polyhedron. Furthermore, the calibration is based in the accelerometer error model, which requires low computation. The proposed method was performed with a low-cost sensor, its effectiveness was compared with similar proposals, and it resulted in higher performance.

## 3. Accelerometer Error Model

A basic mathematical model of an accelerometer is [14]:

(1) $$\tilde{a_{i}}=Sa_{i}+b$$

where *i* represents an axis, i.e., *x*, *y*, and *z*. $\tilde{a_{i}}$ is the accelerometer output, *S* is scale factor, $a_{i}$ is the actual acceleration experienced by the device, and *b* is a constant bias in the measurement. However, this model is too simple and does not reflect imperfections such as cross coupling effects, caused by non-orthogonally mounted axes, and the nonlinearity of the noise in the measurements [14]. A more complete model is [29]

(2) $$\tilde{a_{i}}=\left(1+S_{i}\right)a_{i}+b_{i}+n_{i}$$

where $S_{i}$ is a polynomial that includes nonlinear effects in the sensor and $n_{i}$ is a random bias. $a_{i}$ is the acceleration without considering bias, and it includes the acceleration experienced by the sensor and the influence of the gravity field on the proof-mass [14]. Equation (2) is an adaptation of a model for macro-size accelerometers proposed by Titterton in [30], and it is the accelerometer model considered in this article.

The types of error present in MEMS accelerometers include nonlinearity, random noise, large bias, axes misalignment, and drift [16]. Those errors, when accumulated over time, lead to prohibitive errors in the accelerometer output. The model in (2) try to represent these imperfections, thus its coefficients change according to the conditions present in the sensor. They change over time, applied motion, vibration, temperature, and between power-ons [16].

## 4. Calibration Method

In this section, the calibration method is introduced. It is an extension of the testing plan proposed in [31]. The new elements in this work concerning the testing plan are that the calibration method is based only in three steps that analyze the sensor behavior when stationary and when subject to gravity-related accelerations. The scale factor and bias are estimated by using the accelerometer error model. An algorithm is proposed to remove unintentionally induced accelerations during the evaluations and to identify the samples of interest. Finally, the Allan Variance is used to estimate the terms of velocity random walk and bias-instability.

**Figure 1:**
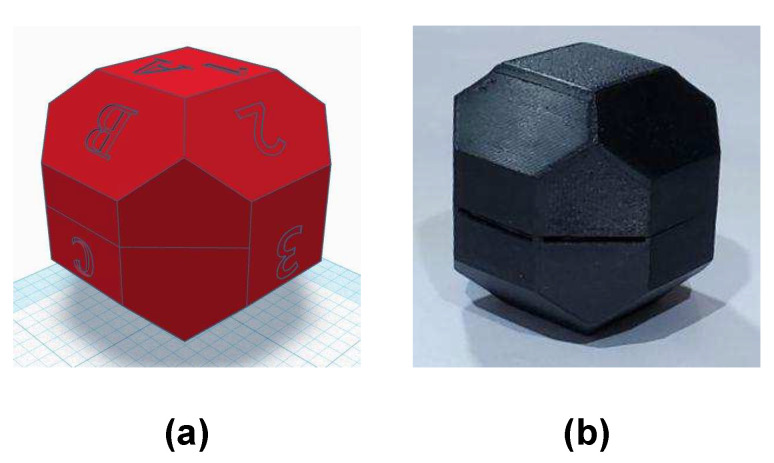
3D printed polyhedron with 18 faces used for a precise positioning of the sensor. (**a**) conception of the polyhedron; (**b**) printed polyhedron.

**Figure 2:**
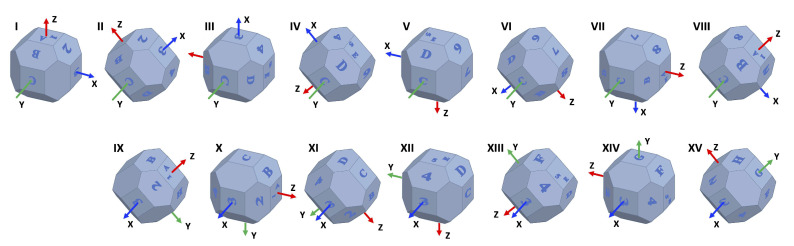
Polyhedron rotating sequence and its effect in the accelerometer axes.

(1) Preliminary Evaluation: In this step, a sanity check is done in the accelerometer, to verify that the sensor performs according to the manufacturer’s specifications. The verification is necessary because the performance of MEMS sensors tends to degrade over time, due to its dependence on the hermetic seal of sensor’s packaging [32]. To perform it, the sensor’s output is observed for a short period of time with the device static; the orientation is not significant. Then, it is verified that the output is within the manufacturer’s specifications.
  Some sensors have a self-test mode, if the accelerometer under calibration has one, it can be used instead. The self-test mode just has to be enabled and the output observed.
  If the accelerometer output is not within the expected values, the sensor is not usable and should not be considered for further evaluations.
(2) Multi-Position Test: The gravity vector is used as reference information to calibrate the sensor in this step. When an accelerometer is static, the only force influencing its proof-mass is gravity; because it is a known value, it can be used to calibrate against it. This step involves to place the sensor static in multiple specific orientations, observe its output and compare it with the gravity value. A polyhedron with 18 faces is used for the precise positioning of the sensor. The solid can be generated with a 3D printer, thus the calibration is accessible and low-cost. In Figure 1, the proposed polyhedron is shown. Figure 1a presents its concept and Figure 1b the printed polyhedron that we used for the calibration described in this article. For the making of the polyhedron, we started with a sphere; then, we performed cuts at every 45 degrees with a depth of 10% of its diameter. The sensor under calibration is attached to one of internal faces. Multiple sensors can be calibrated simultaneously, and the polyhedron can have as many as 14 sensors attached to its inner walls, making the calibration of all of them at the same time possible.
  Verification of the 3D printed polyhedron.
  An important factor of the polyhedron structure is that the opposite faces are parallel to each other. This can be verified by measuring the distance between opposite faces with a Vernier Caliper. Two faces are parallel if the distance between them is the same in every point. Additionally, we recommend printing the solid in two equal-sized parts that assemble horizontally, one above the other, as illustrated in Figure 1b. In this manner, the polyhedron flat faces 1A and 5E will be printed over the printer bed, which is a level surface; thus, possible unevenness in the printed faces are minimized.
  The faces of the polyhedron should be marked with different labels, thus they act as a guide to trace the positions that have already been used. An example of the solid labeling is shown in Figure 1a, where numbers are used in one rotating direction and letters in the other. When performing the calibration, the polyhedron should be put on a rubber mat to minimize any vibration present in the room where the procedure is conducted.
  After attaching the accelerometer inside the polyhedron, the solid is rotated to place the sensor in different orientations. With the proposed polyhedron, those orientations will cause the sensor to experience an acceleration of 0 g, ±0.707 g, and ±1 g. The sensor has to be static on each position for a short period of time; we recommend one minute so the polyhedron can be easily operated, and any noise caused by its manipulation is averaged out of the recorded data [33]. The rotation can start with the 1A face pointing up, followed by rotating in the direction of the labels with numbers, from the smaller number to the largest. After a full rotation, the same process has to be done on the faces marked with letters. When both full rotations have been performed, the accelerations when the sensor was subject to 0 g, −0.707 g, −1 g, 0.707 g, and 1 g have been measured. Figure 2 illustrates the rotating sequence with the polyhedron and the effect that the positions cause in the accelerometer axes.
  The measurements have to be recorded. The data will have certain artifacts caused by the polyhedron manipulation during the rotations. The procedure in Algorithm 1 can be used to remove the accelerations induced by the manipulation and identify the samples of interest. Thereafter, the clean data can be used to find the null bias and scale factor.
  The null bias can be approximated with [30,34]:
  (3) $$B_{i}=\frac{a_{i}^{-up}+a_{i}^{-down}}{2}$$
  where $a_{i}^{-up}$ is the average of $\tilde{a_{i}}$ when the *i* axis is pointing up, $a_{i}^{-down}$ is the average of $\tilde{a_{i}}$ when the *i* axis is pointing down, and $B_{i}$ is the bias term. In an ideal accelerometer, $a_{i}^{-up}$ and $a_{i}^{-down}$ have opposite values, the sum resulting in zero. Any deviation from that value is the sensor bias.
  The scale factor can be similarly found:
  (4) $$S_{i}=\frac{a_{i}^{-up}-a_{i}^{-down}-2g}{2g}$$
  where the numerator should be zero, any difference is $S_{i}$.

**Algorithm 1:**Find the measurements when the sensor was static in the orientations of interest.
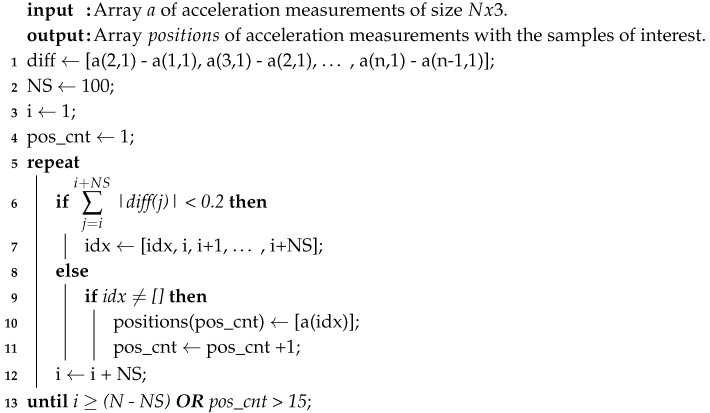

**Figure 3:**
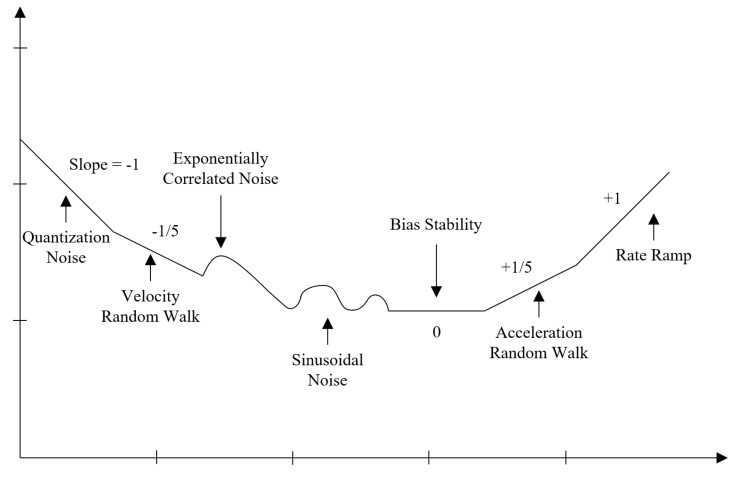
Root Allan variance plot.

(3) Stability Test: In this step, the accelerometer is placed static at a constant temperature for an extended period of time, at least four hours [35]. The acceleration measurements are recorded during this time, and used to determine the velocity random walk and bias instability of each of the sensor’s axis. The Allan Variance [20,21] is applied to estimate them. It is a method to measure frequency stability in oscillators in the time domain, and it can be used to find intrinsic noise in a system as a function of its sampling period. The use of the Allan Variance was firstly proposed in [35], and, because of its simplicity, it has become a popular method for determining the different noise terms that exist in inertial sensor data. Its advantages are the ease of computation and the simplicity in the determination of the source of error; the latter can be achieved through observing slopes variations on the Allan Plot. The results of this step are five basic noise terms appropriate for inertial sensors, they are: quantization noise, velocity random walk, bias instability, acceleration random walk, and rate ramp [11,22]. They are exemplified in Figure 3 [36].
  The Allan Variance, $\sigma^{2}\left(\tau \right)$, can be computed in a data sequence, Ω(t), of length *N* and fixed sample period of Δt, as a function of an averaging time, τ. Through dividing Ω(t) in subsets of consecutive output values, ${\bar{\Omega }}_{k}\left(\tau \right)$, averaged over τ, using the following [22]:
  (5) $$\sigma^{2}\left(\tau \right)=\frac{1}{2(N-2n)}\sum_{k=1}^{N-2n}{\left[{\bar{\Omega }}_{k+1}\left(\tau \right)-{\bar{\Omega }}_{k}\left(\tau \right)\right]}^{2}$$
  (6) $$n=\frac{\tau }{\Delta t}$$
  where
  (7) $${\bar{\Omega }}_{k}\left(\tau \right)=\frac{1}{\tau }\int_{t_{k}}^{t_{k}+\tau }\Omega \left(t\right)dt,\;\Delta \left(t\right)\leq \tau \leq N\Delta t/2.$$
  The confidence in the estimation of the Allan Variance is directly proportional to the number of independent subsets that can be formed from the sampled data. From the Allan Variance, we can get the Allan Deviation through its squared root. After computing a range of different sampling times and plotting it in a Log-Log graph, the different noise components can be identified by observing the gradient changes of the slope [11], as seen in Figure 3. Our main interests are velocity random walk and bias instability.
  Velocity random walk is a high-frequency noise term that appears when the slope has a gradient of −0.5 and the reading is done at τ=1. This is also considered the white noise component of the sensor’s output. Bias instability is located where the gradient is 0. This term, as the name implies, translates into the bias consistency in the measurements changes over a long period of time [4]. The reason why we focus on these two noise terms is because they are a high order integration error [11], and they must be compensated when the sensor is used in applications that require high accuracy, for example, on Inertial Navigation Systems.

## 5. Calibration of a MEMS Accelerometer

The calibration method was applied with the sensor NXP FXOS8700CQ [37], which is a three-axis linear accelerometer and three-axis magnetometer. The accelerometer has a configurable range of ±2 g, ±4 g, and ±8 g, and a resolution of 14 bits. We chose this sensor because it is a very low-power consumption chipset; it consumes only 8 μA in low-power acceleration mode, and 2 μA in standby mode. Additionally, it has low noise density and low output noise range [38]. It is considered as one of the accelerometers with the lowest noise in the low-cost range market.

**Figure 4:**
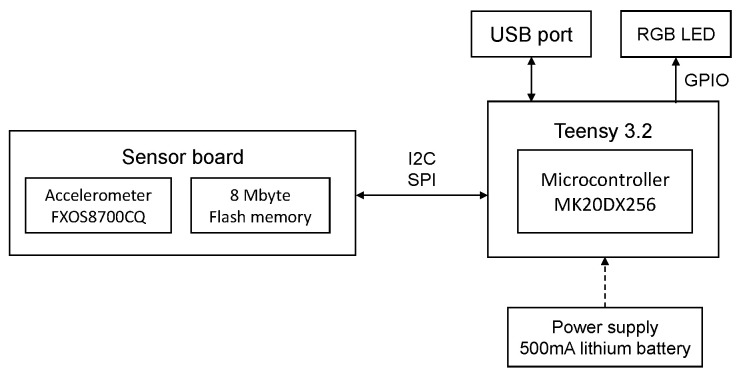
Block diagram of the test bench used for the data capture.

**Figure 5:**
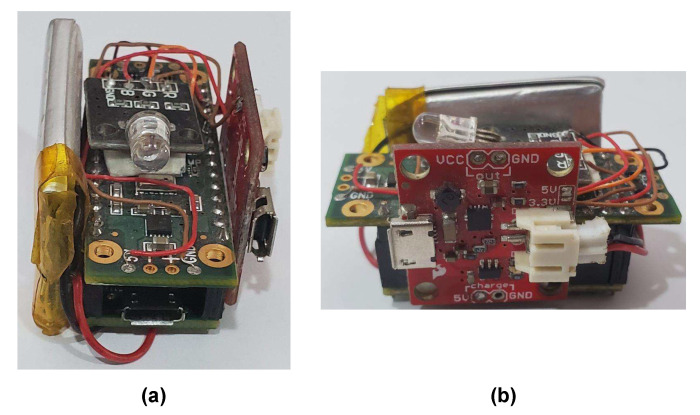
Illustration of the test bench used for the data capture. (**a**) Teensy 3.2 board and Prop Shield sensor board; (**b**) lithium-battery charger module.

The block diagram of the test bench used for the data capture in the accelerometer calibration process is shown in Figure 4. The system consist of a Teensy 3.2 board [39], and a Prop Shield sensor board [40], which contains the FXOS8700CQ accelerometer (NXP Semiconductors, Eindhoven, Netherlands) and a serial 8 Mbyte flash memory. An illustration of the test bench can be seen in Figure 5, where (a) presents the Teensy 3.2 board and Prop Shield sensor board, and (b) the lithium-battery charger module.

As has been previously mentioned, the errors in inertial accelerometers change according to the current conditions on the sensor. Therefore, the following calibration results described below are suitable only for the device under calibration. For other sensors, the same proposed procedure has to be executed on them.

In the target applications of the calibration method, the accelerometer experiments with indoor temperatures. As the sensor is not exposed to extreme temperatures, the sensitivity fluctuation is expected to be low. The temperatures to which the sensor was exposed during the calibration were in the range 24.81 °C to 27.56 °C.

Before calibrating an accelerometer, its current performance has to be known. It can be estimated through computing the magnitude of the acceleration measurements. With an static sensor, the magnitude can be obtained with [25]:

(8) $${\tilde{a_{x}}}^{2}+{\tilde{a_{y}}}^{2}+{\tilde{a_{z}}}^{2}={\left(1g\right)}^{2}$$

**Figure 6:**
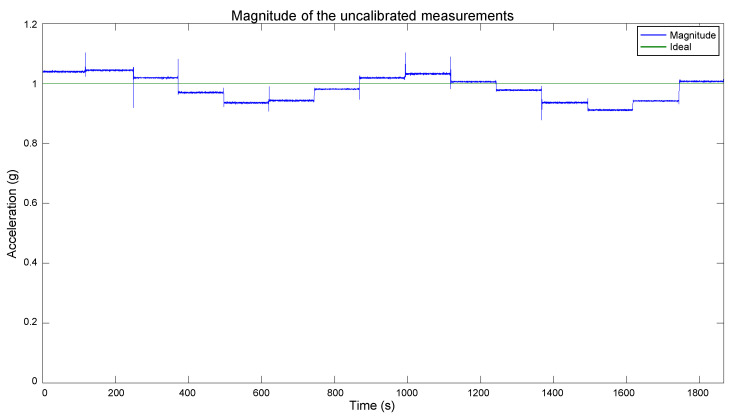
Magnitude of the acceleration measurements of the FXOS8700CQ sensor.

To estimate the performance of the FXOS8700CQ sensor, we measured the accelerations in its three axes while rotating and keeping static the device in different orientations to influence each axis. We recorded the measurements and computed the magnitude according to (8). The resulting magnitude is shown in Figure 6. The short duration spikes that can be seen in the plot correspond to an axis change, and they are caused by the device manipulation and are not significant when examining the sensor current performance. According to (8), a constant magnitude of 1 should be observed. However, in Figure 6, it can be seen that the magnitude deviates from 1. Therefore, the sensor’s axes have a scale and bias problem. Thus, the proposed calibration method will be executed to calibrate the accelerometer output. The next subsections detail the procedure.

### 5.1. Preliminary Evaluation

**Figure 7:**
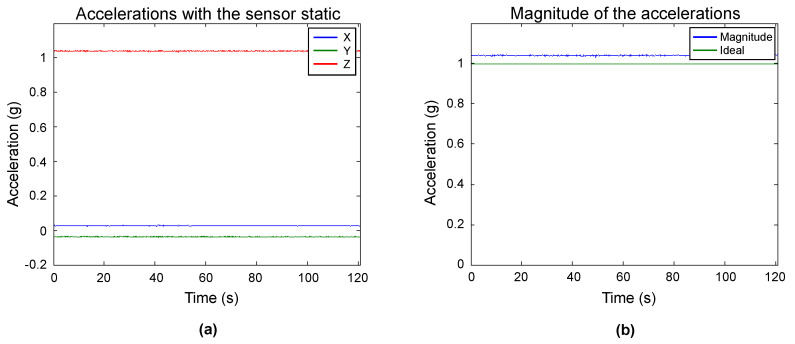
Preliminary evaluation of the sensor. (**a**) measurements when the sensor is static with the *z*-axis pointing to the ground; (**b**) magnitude of the measurements.

This step is a sanity check to confirm that the sensor performs according to its manufacturing specifications. In the FXOS8700CQ, it was executed through positioning the device static with only one axis pointing to ground. The sensor output was recorded, and it can be observed in Figure 7a. From these measurements, we can appreciate that the sensor has a stable behavior, and the output is as expected. Because only one axis is pointing to ground, only one is being influenced by the gravity vector. In Figure 7a, we have the *z*-axis, which detects an acceleration of slightly more of 1 g. No single axis should measure more than 1 g when static, and the error can be attributed to a scale factor in the sensor, which will be compensated with the proposed method. The *x* and *y* axes are measuring near 0 g. Because they are perpendicular to the ground, they are not registering the gravity vector. The offset from 0 g can be attributed to the sensor internal bias.

The magnitude of the samples was obtained to further examine the accelerometer output, and the plot of the magnitude and its expected value can be seen in Figure 7b. A magnitude close to 1 g is expected, which is the case. The sensor has a behavior within its manufacturing specifications; thus, we can proceed to the next steps.

### 5.2. Multi-Position Test

**Figure 8:**
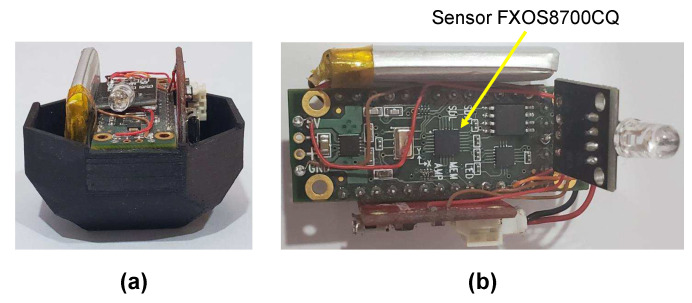
(**a**) small version of the proposed polyhedron; (**b**) location of sensor FXOS8700CQ in the Prop Shield sensor board.

**Figure 9:**
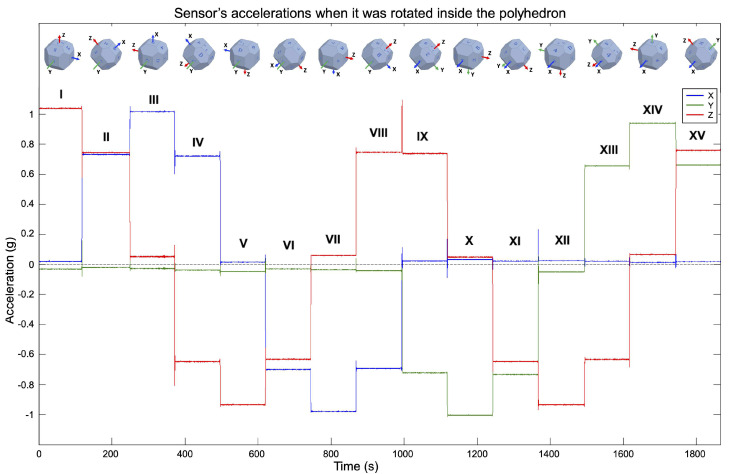
Measurements when the sensor was rotated with the polyhedron. The labels above the measurements are used to differentiate the orientations.

In this step, the sensor is placed static in multiple specific orientations. The polyhedron was used for the precise positioning, as shown in Figure 8. The sensor was attached to an internal face. The solid was continuously rotated and kept static in each position. It was first rotated in the direction of the numbered faces, and after in the ones with letters. The sensor output was recorded at a frequency of 10 Hz. The measurements are shown in Figure 9. Indicators were added to the graph for an easy identification of the measurements of each position. The artifact between the VIII and IX measurements is a minor stop transition when the rotation changed from the labels with numbers to the letters.

A total of 14 different positions can be done with the proposed solid. The full rotation of all the labeled faces results in 15 positions. However, measurements V and XII correspond to the same position; thus, one can be removed. Measurement XII was deleted.

Positions I, III, and X are important because they cause one sensor axis to be aligned with the gravity vector, and the other two to be orthogonal to it. Thus, a read of ±1 g is expected on one axis and 0 g in the rest. In the positions V, VII, and XIV, the sensor has the opposite orientation with respect to I, III, and X. The positions pair I and V submit the sensor *z*-axis to 1 g and −1 g, respectively, and the *x*- and *y*-axes to 0 g. Positions III and VII submit the sensor *x*-axis to 1 g and −1 g, respectively, and *y* and *z* axes to 0 g. Finally, the positions X and XIV cause the *y*-axis to experience −1 g and 1 g, respectively, and *x*- and *z*-axes to have 0 g. The pairs I–V, III–VII, and X–XIV are, therefore, used to estimate the scale factor and bias terms. The rest of the positions are convenient for evaluating the calibration results because they submit two axes to a fraction of the gravity vector (±0.707 g).

**Figure 10:**
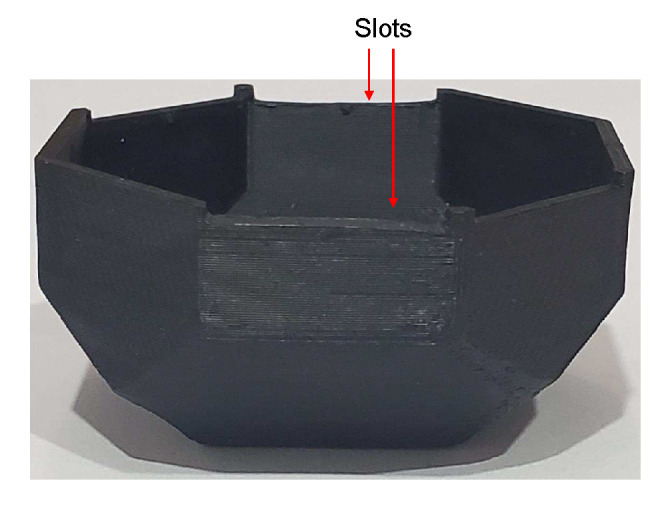
Slots in one-half of the polyhedron to hold and level the test bench.

The pairs III–VII, X–XIV, and I–V will be used to calibrate the *x*-, *y*-, and *z*-axes, respectively. However, this is dependent on how the sensor is mounted in the polyhedron. For determining the calibration parameters, the sensor must be mounted parallel to one of the labeled faces, and one of its axes has to be aligned with the rotating direction, i.e., one axis should be pointing to one of the adjacent labeled faces. To replicate the results showed in this article, the configuration that we used was: the sensor’s *x*-axis pointed to label “3”, *y*-axis to “G”, and *z*-axis to “1”. To ensure that the sensor is mounted parallel to a labeled face and one of its axes is aligned with the rotating direction, we printed in one-half of the polyhedron two horizontal slots to hold and level the test bench. The slots are opposite to each other, parallel to the flat faces, and over two labeled faces. They were an optimization to minimize the print size of the polyhedron used for the calibration and were a custom addition suitable to the test bench size. Otherwise, each face of the solid would have to be of the test bench size. In Figure 10, we can see the slots in the polyhedron used in the calibration.

**Figure 11:**
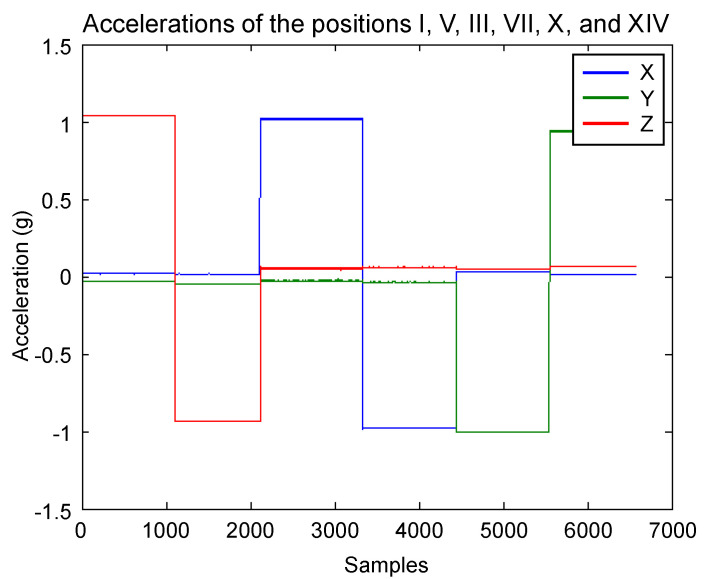
Measurements of the key positions after removing artifacts caused by the device manipulation.

Algorithm 1 was applied to the measurements to remove artifacts caused by the manipulation of the solid. The resulting data of the key positions are shown in Figure 11. With these data, null bias and scale factor can be found using (3) and (4). In Table 1, the averages of the key position measurements are displayed for each axis. Afterwards, they are used to determine the null bias and scale factor, which are shown in Table 2.

### 5.3. Stability Test

**Figure 12:**
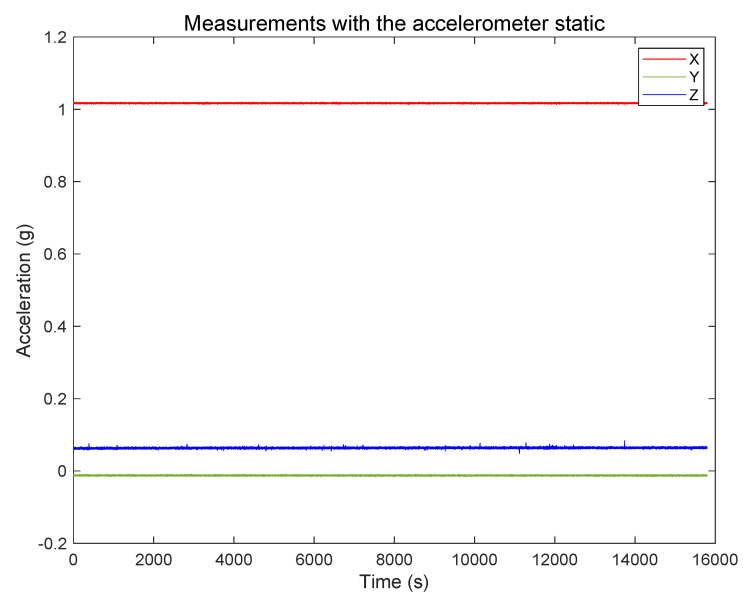
Measurements with the sensor static for 4.4 h.

This step consists in positioning the sensor static for several hours at constant temperature and record the acceleration measurements of the three axes. The Allan Variance is applied in the measurements to estimate noise terms present in inertial sensors. In the test, we positioned the sensor static for 16,000 s (4.4 h), and we recorded the accelerations at a frequency of 2 Hz. The data are shown in Figure 12. The extended measurements enable the computation of the velocity random walk and bias instability of each axis, which are high order integration errors.

**Figure 13:**
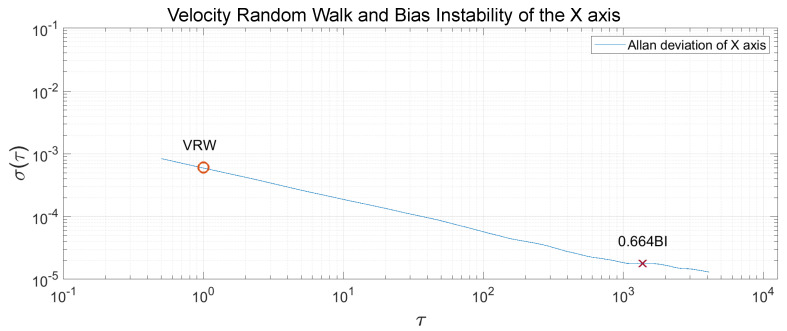
Velocity random walk and bias instability of the sensor *x*-axis.

**Figure 14:**
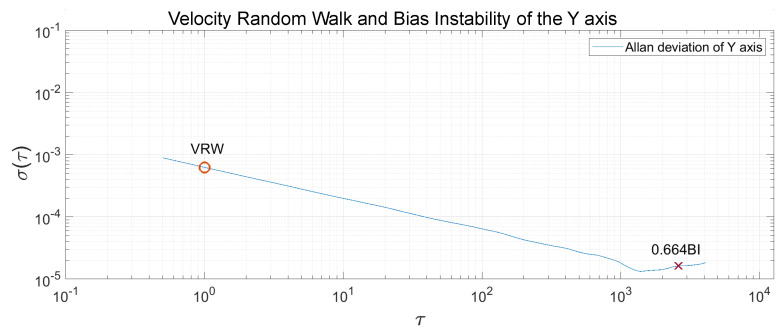
Velocity random walk and bias instability of the sensor *y*-axis.

**Figure 15:**
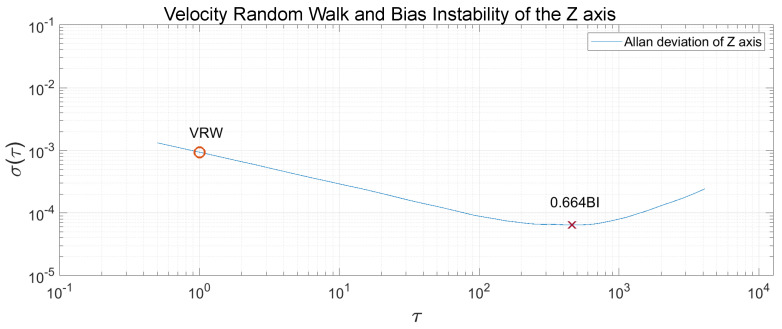
Velocity random walk and bias instability of the sensor *z*-axis.

The Allan Variance was computed in the recorded measurements, and, after that, the Allan Deviation through the squared root. The noise terms of velocity random walk and bias instability were obtained through observing the gradient changes of the slope in the Log-Log plot of the Allan Deviation. Velocity random walk is located when the slope has a gradient of −0.5, and the reading is done at τ=1. Bias instability can be obtained where the gradient is 0, scaled to $\sqrt{\frac{2ln2}{\pi }}\approx 0.664$ [22]. In Figure 13, Figure 14 and Figure 15, the velocity random walk (VRW) and the bias instability (BI) of each of the sensor axes are represented. Their values are shown in Table 3.

### 5.4. Results

**Figure 16:**
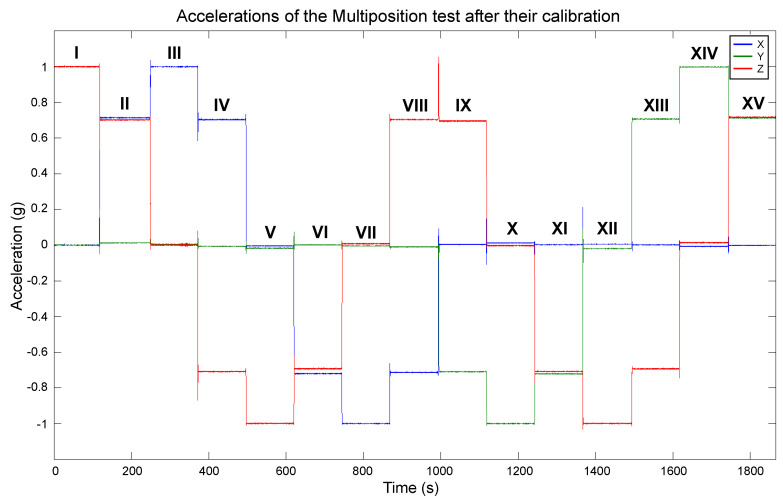
Measurements of the sensor rotation with the polyhedron after their calibration.

**Figure 17:**
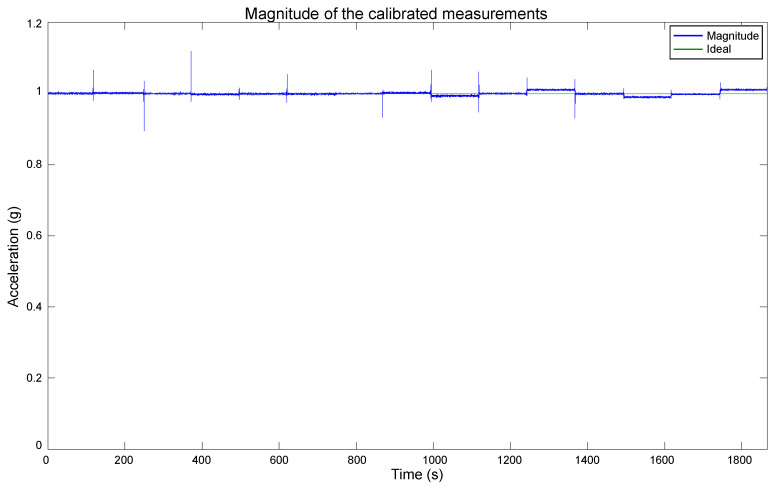
Magnitude of the measurements after the calibration.

The estimated bias and scale factor were used to compensate the errors in the measurements utilizing (2). The compensation was performed on the measurements obtained with the polyhedron in the Multi-position test, which were presented in Figure 9. We chose those measurements for the calibration because they contain diverse accelerations for each axis, and their actual values are known. The compensation resulted in the calibrated data shown in Figure 16. As mentioned in the beginning of this section, the short duration spikes are caused by the polyhedron manipulation, and they do not affect the evaluation of the calibration performance. In Figure 16, it can be appreciated that the data are very close to the expected behavior. Each axis has a maximum and minimum value of 1 g and −1 g, respectively, and their null value is close to the 0 mark. Therefore, these calibration values have been approximated correctly, a vast improvement over the original data shown in Figure 9. To compare the calibration results with the original data, the magnitude of the calibrated data was computed, as shown in Figure 17. As can be seen, a magnitude of 1 g is achieved for most of the measurements, significantly improving the obtained magnitude in Figure 6.

**Figure 18:**
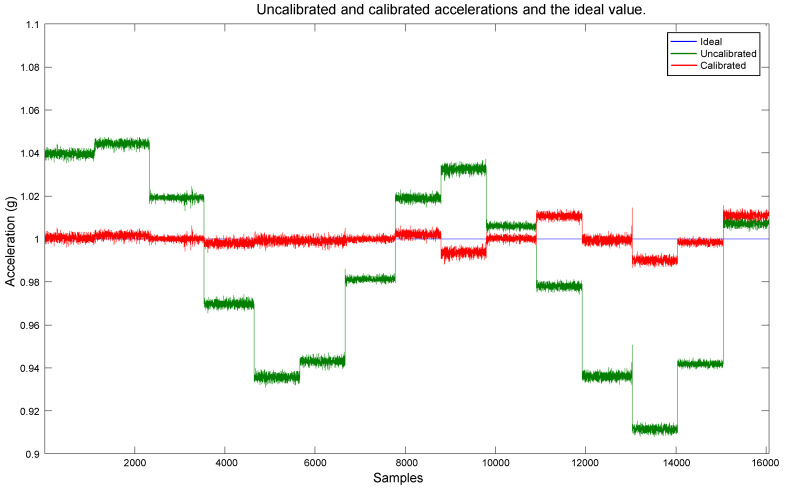
Comparative between the uncalibrated and calibrated data, and the ideal value.

A comparative between the magnitude of the uncalibrated and calibrated measurements, and the ideal value is presented in Figure 18. For a clearer view of the calibration results, the spikes caused by the manipulation of the polyhedron when rotating the sensor were removed, and the plot scale was zoomed in.

Small deviations from the ideal value can be observed in the last positions of the plots in Figure 16, Figure 17 and Figure 18. They can be caused by a few factors.

1. Misalignments in the vertical faces of the polyhedron caused by minor defects in the printing.
2. Misalignments with the two halves of the polyhedron. This is a shortcoming of the current computer-aided design (CAD). Adding snap-on aligning pins to the polyhedron is on the future improvement list.
3. Finally, movements of the sensor related to the printed object while it is being rotated. This is also a future mechanical improvement to the CAD, in order to achieve a firm sensor positioning with the printed object.

## 6. Discussion

In this paper, a low-cost method for accelerometer calibration is proposed, which consists of three steps very simple to perform. In the first one, it is verified that the sensor is usable, through confirming that it performs within the manufacturer specifications. The importance of conducting this step is because accelerometers are prone to performance degradation over time, due to their dependence on the hermetic seal of their packaging. The second step consists in positioning the sensor static in specific orientations where the experienced acceleration is known. When a device is static, gravity is the only force influencing its axes; thus, the acceleration in those orientations is related to the gravity vector. To be able to position the sensor in the precise orientations, a 3D printed polyhedron was used. The solid has 18 faces and the sensor is attached to an internal one. The polyhedron is rotated in two directions to position the accelerometer in 14 orientations where the acceleration force is of 0 g, ±0.707 g, and ±1 g. The measurements are recorded during the rotations. The null bias and scale factor are approximated from this data, through a simple relationship between the measurements and the actual acceleration value. The last step of the proposal consists in placing the sensor static at a constant temperature for an extended period of time. Then, velocity random walk and bias instability of each of the sensor’s axis are determined, through computing the Allan Variance in the measurements. It is a mathematical method with low computing requirements, which can help to find the intrinsic noise in a system as a function of its sampling period. The method has been used to find noise terms in inertial sensor measurements. Velocity random walk considers the white noise component of the sensor output. Bias instability is the bias consistency in the measurements changing over a long period of time. We focused on these two noise terms because they are a high order integration error.

The proposed calibration method was performed with the low-cost sensor NXP FXOS8700CQ, which contains a three-axis linear accelerometer. It is a very low-power consumption chipset, and is considered as one of the accelerometers with the lowest noise in the low-cost range market. For the evaluation of the calibration, the magnitude of the acceleration measurements with the sensor static was obtained, before and after the calibration. When an accelerometer is static, gravity is the only force influencing its axes; thus, a magnitude of 1 g should be obtained. To evaluate the error present in the measurements, the RMSE of each magnitude was computed. In Table 4, these values are presented. The percentage of error reduction due to the calibration with the proposed method is 88.46%. As can be seen, the proposal decreased the error in the measurements significantly—thus increasing the accuracy in the sensor’s output. The RMSE of the calibrated measurements was compared with the reported in similar proposals, including those of the authors Mulloy et al. [23], Won et al. [25], Ghaffari et al. [41], and Lee et al. with the methods of Recursive Least Squares (RLS) and Fourier Transform (FT) [42]. Their RMSE values and the difference with ours are shown in Table 5. As can be seen, the proposal has a RMSE significantly lower than the comparing methods. The larger difference is of 0.0447 with the work of Lee et al. (RLS), and the smallest of 0.0041 with Won et al.

To have a precise calibration, the polyhedron structure must not have imperfections, and the surface where the calibration is performed must be level. However, we consider that these requirements are feasible. With the printing precision and tolerance of 3D printers at the present day, printing errors such as incorrect dimensions or unevenness are minimal. Furthermore, the errors can be easily detected by measuring the polyhedron faces and can be corrected by smoothing the polyhedron surface. Concerning the requirement of performing the calibration on a flat surface, the surface level can be verified at low cost with a digital level tool or a digital angle gauge, and the surface can be adjusted according to the measurements indicated by the tools. We consider that the advantages of our proposal, which include low cost, user-friendly, low computational cost, and high reduction of errors in measurements, outperform the disadvantages of meeting the method’s requirements.

## 7. Conclusions

In this article, a simple, practical, and low-cost calibration method for low-cost MEMS accelerometers has been presented. To accomplish these characteristics, the method does not use any sophisticated equipment as it is done in other proposals. Instead, it uses only a 3D printed polyhedron, benefiting from the popularization and low-cost of 3D printing in the present day. Each polyhedron can hold multiple sensors at the same time, and all of them can be calibrated simultaneously. As the polyhedron is small and light, it can be transported to any place to perform the calibration. Furthermore, the method is simple; thus, it can be done in any place and moment, not only by experts in laboratories.

The proposal is comprised of three steps. They consist of a sanity check, a multi-position evaluation, and a stability test. The accelerometer error model was used for the error compensation; thus, the computational requirements are very low. The proposed method was performed with the low-cost sensor NXP FXOS8700CQ (NXP Semiconductors, Eindhoven, Netherlands). To evaluate the calibration results, the magnitude of the acceleration measurements with the sensor static was obtained, before and after the calibration. The RMSE of each magnitude was computed and then compared, which showed that the proposed method decreased the error in the measurements by 88.46%—thus increasing significantly the accuracy in the sensor’s output. The RMSE of the calibrated data was also compared with the reported in similar proposals, and our method resulted in higher performance.

The simplicity and low-cost of the proposed method enables a practical calibration for low-cost MEMS accelerometers. The low RMSE of the calibrated data shows that the proposal effectively compensates the accelerometer’s inherent errors, increasing significantly the accuracy of its output.

Future work involves the calibration of accelerometers in smart-phones, as they are one of the devices with higher presence in the consumer market. The same method proposed in this article is suitable for their calibration; however, the design of a new polyhedron is needed to accommodate the size and weight of the device.

## Figures and Tables

**Table 1:** Averages of the six key positions.

	x	y	z
$a_{i}^{-up}\left(g\right)$	1.0175	0.9394	1.0389
$a_{i}^{-down}\left(g\right)$	−0.9788	−1.0043	−0.9343

**Table 2:** Null bias and scale factor for each axis.

	x	y	z
$B_{i}\left(g\right)$	0.0193	−0.0324	0.0523
$S_{i}$	−0.0018	−0.0281	−0.0134

**Table 3:** Velocity random walk and bias instability for each axis.

	x	y	z
VRW ($m/s/\sqrt{h}$)	$0.6047\times 10^{-3}$	$0.6275\times 10^{-3}$	$0.9278\times 10^{-3}$
BI (mg)	0.02679	0.02461	0.09704

**Table 4:** RMSE of the measurements before and after executing the proposed calibration method.

Measurements	RMSE
Uncalibrated	0.0442
Calibrated with the proposed method	0.0051

**Table 5:** RMSE comparison of the proposal with similar methods.

Method Proposed By	Method’s RMSE	Difference With Our RMSE
Mulloy et al. [23]	0.0215	0.0164
Won et al. [25]	0.0092	0.0041
Ghaffari et al. [41]	0.0139	0.0088
Lee et al. (RLS) [42]	0.0498	0.0447
Lee et al. (FT) [42]	0.0456	0.0405
